# Oxidative stress response to acute hypobaric hypoxia and its association with indirect measurement of increased intracranial pressure: a field study

**DOI:** 10.1038/srep32426

**Published:** 2016-08-31

**Authors:** Giacomo Strapazzon, Sandro Malacrida, Alessandra Vezzoli, Tomas Dal Cappello, Marika Falla, Piergiorgio Lochner, Sarah Moretti, Emily Procter, Hermann Brugger, Simona Mrakic-Sposta

**Affiliations:** 1 EURAC Institute of Mountain Emergency Medicine, Bolzano, Italy; 2 Department of Biomedical Sciences, University of Padova, Padova, Italy; 3 CNR Institute of Bioimaging and Molecular Physiology, Segrate (Milano), Italy; 4 Department of Neurology, General Hospital of Bolzano, Bolzano, Italy; 5 Department of Neurology and Psychiatry, Sapienza University, Roma, Italy; 6 Department of Neurology, University of the Saarland, Homburg, Germany

## Abstract

High altitude is the most intriguing natural laboratory to study human physiological response to hypoxic conditions. In this study, we investigated changes in reactive oxygen species (ROS) and oxidative stress biomarkers during exposure to hypobaric hypoxia in 16 lowlanders. Moreover, we looked at the potential relationship between ROS related cellular damage and optic nerve sheath diameter (ONSD) as an indirect measurement of intracranial pressure. Baseline measurement of clinical signs and symptoms, biological samples and ultrasonography were assessed at 262 m and after passive ascent to 3830 m (9, 24 and 72 h). After 24 h the imbalance between ROS production (+141%) and scavenging (−41%) reflected an increase in oxidative stress related damage of 50–85%. ONSD concurrently increased, but regression analysis did not infer a causal relationship between oxidative stress biomarkers and changes in ONSD. These results provide new insight regarding ROS homeostasis and potential pathophysiological mechanisms of acute exposure to hypobaric hypoxia, plus other disease states associated with oxidative-stress damage as a result of tissue hypoxia.

Failure to adequately adapt to hypobaric hypoxia during rapid ascent to high altitude can result in hypoxemia and tissue oxidative stress caused by an increased production of reactive oxygen species (ROS)^1^. Electron paramagnetic resonance (EPR) is the only technique that provides direct evidence and an absolute quantification of ROS^2^. Oxidative stress quantification is based on the determination of specific end products of the damage resulting from the interaction of ROS and biological macromolecules. Lipid peroxidation is a well-established mechanism of cellular injury and is used as an indicator of oxidative stress in cells and tissues^3^. Inadequate acclimatisation to hypobaric hypoxia can lead to a number of neurological complications, ranging from mild acute mountain sickness (AMS) to high-altitude cerebral edema (HACE). AMS and HACE typically develop within hours to days among lowlanders exposed to high altitude. The etiology of the cerebral edema associated with AMS and HACE can be extracellular (vasogenic) and/or intracellular (cytotoxic)^4^,^5^. Oxidative stress can cause extracellular edema through direct peroxidative stress to the blood-brain barrier (BBB) microvascular endothelium, neurons, and glia^6^. Equally, intracellular edema can be increased due to the promotion of membrane cell swelling through inhibition of Ca^2+^-pump and Na^+^/K^+^-pump ATPase^7^. Normobaric hypoxia reportedly increases blood, lumbar cerebrospinal fluid (CSF) and the net cerebral output of oxidative stress biomarkers^8^,^9^. An increase in oxidative stress was also observed under hypobaric hypoxic conditions correlated with symptoms of AMS^10^. Nevertheless, oxidative stress implications for the pathogenesis of AMS and HACE^5^,^11^ have yet to be fully elucidated. During an acute (18 h) normobaric hypoxic exposure, the increase in ROS production associated with a concomitant increase in brain volume was not associated with gross BBB dysfunction and increased lumbar CSF pressure^10^. Unfortunately, most of the gold standard neuroimaging techniques cannot be performed in the field for practical and ethical issues, limiting the elucidation of the underlying pathophysiological mechanisms. Portable ultrasound devices allow non-invasive evaluation of many neuroimaging parameters of interest during high altitude research. Ultrasonography of the optic nerve sheath diameter (ONSD) has been shown to be a reliable tool for diagnosis of increased intracranial pressure (ICP) in a hospital setting in neurocritical patients^12^,^13^, and ONSD changes have previously been associated with AMS and HACE^14^,^15^,^16^. Despite the assumption that ONSD reflects changes in brain volume, the pathophysiological and clinical significance of increased ONSD in subjects exposed to hypobaric hypoxia is still unclear.

In this study we report the changes over time of ROS production, oxidative-stress biomarkers (for lipid peroxidation damage) and ONSD measurements induced by acute exposure to hypobaric hypoxia in lowlanders. Confounding factors (e.g. physical effort, changes in altitude or diet) were avoided as much as possible. Therefore, we investigated the possible relationship between changes in ROS production, oxidative-stress biomarkers and ONSD.

## Results

### Basic physiological changes to hypobaric hypoxia exposure

Table 1 depicts data acquisition at each medical examination. There was a progressive increase in heart rate (HR) after exposure of healthy lowlanders to high altitude (3830 m), an expected cardiopulmonary adjustment to the unavoidable reduction in oxygen availability. The related decrease of peripheral oxygen saturation (SpO_2_) confirmed the reduction in inspired oxygen partial pressure (barometric pressure 485 mmHg [64.66 kPa]; oxygen partial pressure 102 mmHg [13.6 kPa]). Lake Louise Score (LLS) was used for the diagnosis of AMS and increased in line with duration of hypoxic exposure, however the changes were not statistically significant. Two participants developed AMS (LLS 5 and 9), one of whom had to be evacuated after only 24 h.

### Oxidative stress response to hypobaric hypoxia exposure

**Figure 1:**
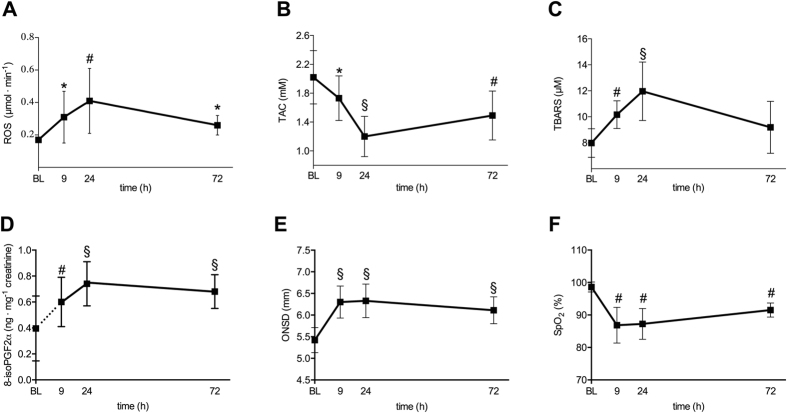
Passive exposure to 3830 m is associated with increased oxidative stress and optic nerve sheath diameter. Time course of mean reactive oxygen species production rate (ROS, μmol∙min^−1^) (**A**), total antioxidant capacity (TAC, mM Trolox) (**B**), thiobarbituric acid-reactive substances (TBARS, μM) (**C**), 8-isoprostanes (8-isoPGF2α, ng∙mg^−1^ creatinine) (**D**), optic nerve sheath diameter (ONSD, mm) (**E**) and oxygen saturation (SpO_2_, %) (**F**) at baseline (BL, 262 m) and after 9, 24, and 72 h at 3830 m. Vertical bars represent standard deviation. P-values refer to different time points compared to baseline and are represented with symbols (*0.01 ≤ p < 0.05; ^#^0.001 ≤ p < 0.01; ^§^p < 0.001).

ROS production rate (determined by EPR assessment), thiobarbituric acid-reactive substances (TBARS), 8-isoprostanes (8-isoPGF2α) and plasmatic total antioxidant capacity (TAC) (determined by immuno and/or enzymatic assays) for the whole examined cohort are shown in Table 1. Oxidative stress biomarkers at baseline (262 m) were in agreement with previous values estimated for a healthy population^17^. Hypobaric hypoxia exposure lead to an increase of indices of oxidative stress; namely, increased concentrations of ROS production, TBARS, 8-isoPGF2α (lipid oxidation products and potential disease mediators), plus a decrease in TAC (the sum of aqueous and lipid soluble low-molecular weight antioxidants). ROS production rate, TBARS and 8-isoPGF2α concentration changed over time of exposure to hypobaric hypoxia portraying a parabolic-like pattern within 72 h (ANOVA p < 0.001, p < 0.001 and p < 0.015, respectively) (Fig. 1); changes were detectable after 9 h and reached a peak of +141% (ROS), +50% (TBARS) and +85% (8-isoPGF2α) after 24 h (p = 0.004, p < 0.001, p < 0.001 compared to baseline) (Table 1). TAC values showed a specular pattern within 72 h (ANOVA p < 0.001) with a nadir of −41% after 24 h (p < 0.001).

### ONSD response to high altitude exposure

ONSD at baseline were in agreement with previous values estimated for healthy population ONSD^18^. In response to acute, passive exposure to 3830 m, ONSD also depicted a parabolic-like pattern within 72 h (ANOVA p < 0.001); changes were detectable within 9 h and reached a maximum increase of +17% after 24 h (p < 0.001).

### Relationships between oxidative stress parameters and ONSD changes

**Figure 2:**
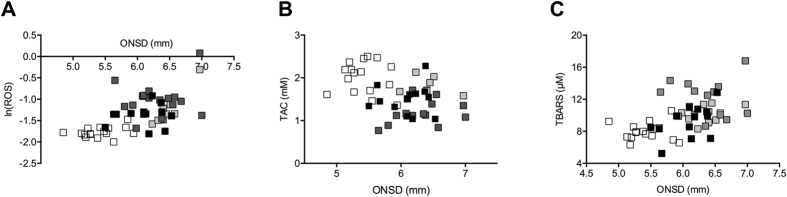
Relationship between oxidative stress and changes in optic nerve sheath diameter (ONSD). At the bottom of the figure, the scatterplots show absolute values of reactive oxygen species (ROS) (**A**), total antioxidant capacity (TAC) (**B**) and thiobarbituric acid-reactive substances (TBARS) (**C)** with optic nerve sheath diameter (ONSD). Empty, light grey, grey and full black squares represent values at baseline and after 9, 24 and 72 h at 3830 m, respectively.

Ln(ROS), TAC and TBARS were correlated with SpO_2_ (p < 0.001 for all tests) (Table 2) and ONSD (p < 0.001 for all tests) (Table 2 and Fig. 2). The correlation of SpO_2_ with ln(ROS) was only marginally significant when time was added to the model (p = 0.081), whereas conversely ONSD was not correlated with ln(ROS) when time was added to the model (p = 0.559) (Table 2). Similarly, no correlation of TBARS and TAC with SpO_2_ and ONSD was found when time was added to the model. The correlation of the three dependent variables; namely, ln(ROS), TBARS and TAC with time was significant in both the SpO_2_ model (p < 0.001, p < 0.001 and p = 0.001, respectively) and the ONSD model (p < 0.001, p = 0.001 and p = 0.036). When adding additional variables, such as: age, body mass index (BMI), gender, percentage body fat or smoker (yes/no) to the ONSD and time model only time remained significant. The correlation analysis of the relative increase from the previous time-point in ROS and TBARS showed similar results (i.e., a correlation with time but not with ONSD and SpO_2_). The correlation analysis of the relative increase in TAC was not significant.

The regression analysis (generalised estimating equations) did not show a significant influence of ROS, TBARS and TAC on ONSD (Table 3).

## Discussion

We report, for the first time, the time course of changes in ROS, TBARS, TAC and 8-isoPGF2α over 72 h of exposure after passive ascent to constant hypobaric hypoxia (3830 m). The observation time of 72 h is consistent with the acute phase of acclimatisation that occurs within the first three days. Changes in oxidative-stress biomarkers were independently correlated with time of exposure even if anthropometric characteristics (age, BMI, gender, and percentage body fat) were considered in the statistical model. Since confounding factors (e.g., physical effort, changes in altitude, diet and tent temperature during examination and blood collection) were minimised, we infer that the increase and subsequent decrease in biomarkers of oxidative stress may reflect the time-related response to acute hypobaric hypoxia. Despite the observation that ONSD showed a similar parabolic-like pattern within 72 h, changes in oxidative-stress biomarkers were not identified as an independent contributor to changes in ONSD in the multivariate analysis.

Hypoxia triggers several cellular processes and responses through different pathways and can produce an imbalance between oxidative stress and antioxidant capacity. Oxidative stress and cellular accumulation of ROS play a vital role in the stimulation of autophagy under conditions of hypoxia, ischemia/reperfusion injury, and other cellular stress responses^19^. There is some evidence from cell and tissue experiments that acute hypoxia induces accumulation of ROS in cells and tissues over time^10^,^20^. This seems to be supported by our results, showing a progressive increase in ROS within 24 h. The prolonged imbalance between ROS production and antioxidant scavenging induction indicates the appearance of oxidative stress-related damages in DNA, proteins and lipids^17^,^21^. Biological membranes constitute, in fact, a preferential target for ROS, largely because of their unsaturated fatty acid content. In our study TBARS and 8-isoPGF2α (end markers for lipid peroxidation damages) increased during the first 24 h of exposure to hypobaric hypoxia, while TAC was downregulated.

Oxidative stress-related damage can have important systemic implications^22^, particularly on cellular structures and transmembrane ion channels in the brain. Despite that in this study the main sites of ROS production and antioxidant compound oxidation (the biological sources of the lipid oxidation products) remain unclear, plasma values peaked in parallel within 24 h of exposure to hypobaric hypoxia; thus indicating an acute oxidative stress. However, previous investigation of ROS production has reported concentration changes assessed in biological samples (blood and urine) seem to adequately reflect variations in tissue of interest (i.e. cerebral tissue)^23^. Neuronal tissue has a high O_2_ demand/consumption and is characterised by a poor concentration of antioxidants but rich concentration of polyunsaturated fatty acids and biological macromolecules, both of which are susceptible to oxidation^24^,^25^. Damage due to oxidative stress-mediated lipid peroxidation can involve the peripheral nerve glia (Schwann cells)^24^. Schwann cells, also be referred to as neurilemma cells, play a vital role in maintaining the peripheral nervous system (PNS) through the production of the protective myelin sheath around neuronal axons. Retinal ganglion cells (RGC) are myelinated long-axon neurons in the central nervous system that constitute the optic nerves. Unlike other neurons, the blood supply of RGC axons is different from those supplying cell bodies^26^, potentially making them particularly sensitive and vulnerable to different types of stress-induced damages; including oxidative stress. Moreover, the large number of mitochondria in RGC axons potentially expose them to a greater increase in ROS during oxidative stress^27^. Hence our particular interest in ONSD in relation to an acute hypobaric hypoxic stressor.

Consequently, although the main objective of our study was to assess the temporal and quantitative changes of the oxidative stress biomarkers in adaptation during 72 h of exposure to hypobaric hypoxia, several intriguing associations between biochemical parameters, physiological variables, neuroimaging parameters and hypoxia-related oxidative stress response are worthy of further investigation. Our results do not support an independent relationship between markers of oxidative stress, oxidative stress-related damages and changes in ONSD after acute exposure to hypobaric hypoxia. However, we cannot exclude a contribution of oxidative stress-related damages with only a marginal effect on ONSD because of the small sample size of our study. Clearly, additional mechanistic investigations are warranted to identify the sources of ROS production (i.e., whether they originate in blood cells, neuronal or vascular tissue, skeletal muscle tissue or are derived from other organs), the sequence of signalling pathways involved, and consequential relationships with phenotypic tissue changes.

Previous investigation of ONSD showed a different time course of changes in ONSD in those patients who developed AMS compared to the subjects who did not^15^; subjects with AMS (and HACE) had a larger ONSD than those without^18^. Despite these findings and the fact that ONSD ultrasonography has been used under normobaric conditions to monitor intracranial pressure (ICP) in patients with brain injuries of different pathogenesis (e.g. brain trauma, intracranial bleeding)^12^,^13^ and inflammation of the nerve itself^28^, underlying pathophysiological mechanisms causing changes in ONSD under hypobaric hypoxia haven’t been investigated yet. Our results do not seem to suggest a direct causal relationship between oxidative stress and the enlargement of ONSD in hypobaric hypoxia. This is in agreement with the lack of change reported in S100b, NSE, CSF–blood protein concentration quotients in relation to the intended induction of oxidative stress in a previous 18-h normobaric hypoxic study^8^. The measurement of ONSD seems to simply reflect a general rise in brain volume in subjects exposed to hypobaric hypoxia^11^. Despite that no direct comparison between brain volume, ICP and ONSD in hypobaric hypoxia has been made so far, a relative increase in ICP (although within the normal range) is suggested by the increase in grey and white matter volumes (and a decreased in CSF) after 10 and 22 h of normobaric hypoxia in MRI studies^29^,^30^. The same effect on ICP was suggested by different in-field studies with other neuroimaging techniques^11^.

One limitation of the study is the lack of determination of biomarkers for increased permeability and inflammation in CSF, due to operative field conditions. Secondly, our conclusion is not based on casual effects from observations but on a lack of association. The small sample size (as in most prospective altitude studies) cannot exclude a type II error, despite using more time-points for observation and reducing potential confounding factors as much as possible.

## Conclusion

This 72 h in-field study describes changes of oxidative-stress biomarkers during passive hypobaric hypoxia exposure in lowlanders for the first time. The lack of association of oxidative-stress biomarkers with ONSD changes seems to exclude a significant direct causal relationship, but further studies with neuroimaging techniques under natural and simulated hypoxic conditions are necessary to better elucidate the clinical significance of increased oxidative stress biomarkers and ONSD during exposure to acute hypobaric hypoxia.

## Materials and Methods

### Experimental design

**Figure 3:**
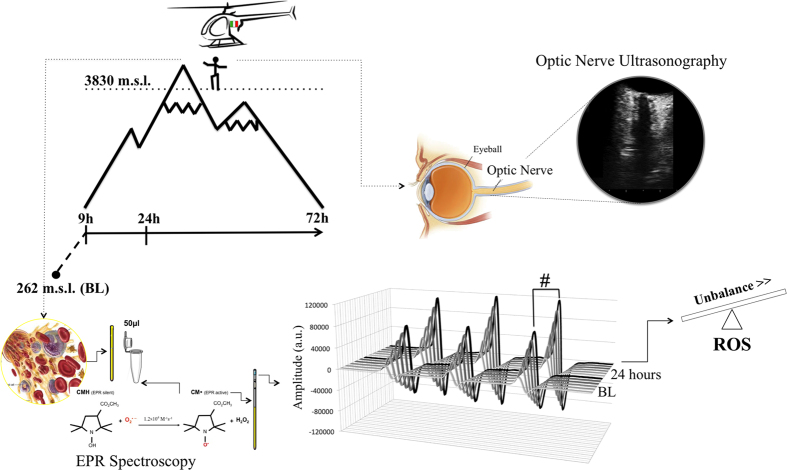
Study design and clinical and biological measurements. Sketch and chart indicate exposure profile and timing of blood sampling and optic nerve sheath diameter (ONSD) measurements. At right in the upper figure, the optic nerve ultrasonography scan with hypoechogenic ocular globe and a longitudinal section of optic nerve. Measurements of ONSD were done 3 mm behind the papilla using an electronic caliper and an axis perpendicular to the optic nerve. At the bottom of the figure the blood sample preparation and electron paramagnetic resonance (EPR) acquisition protocol. CMH Spin Probe (50 μl) was added in equal amount (1:1) to the collected blood. The solution was immediately put in a glass EPR tube. From the generated radical compound, in the time course of the reaction, ten EPR spectra were collected in about 6 min, one of which is shown. The signal amplitude (a.u.) is proportional to the number of paramagnetic spin formed at the acquisition time. The calculated rate production values was converted in absolute levels (μmol·min^−1^) by using CP^●^ radical as external standard.

Sixteen Caucasian participants (4♀, 12♂) were recruited. Mean age was 39 ± 10.2 yr, weight 71.6 ± 8.7 kg, height 173.3 ± 6.8 cm, BMI 23.8 ± 2.2 kg/m^2^, percentage body fat 23.2 ± 5.5% and three of the sixteen participants were smokers. Exclusion criteria included: habitual intake of antioxidant or anti-inflammatory substances at baseline visit; acute illness (infectious, cardiovascular, cerebrovascular or respiratory); ocular pathology; and prior acute high-altitude illness. Participants were examined at baseline (262 m) and after passive ascent by helicopter to 3830 m (9, 24 and 72 h) (Fig. 3). Participants remained without physical effort at constant altitude (barometric pressure 485 mmHg [64.66 kPa]; oxygen partial pressure in 102 mmHg [13.6 kPa]) and they adhered to a standardised food intake. Medical examinations including SpO_2_, HR, LLS and blood collection were performed in a heated tent at a controlled temperature. AMS was defined as LLS ≥ 3 with headache^31^. The study was approved by the local Ethics Committee of Bolzano (0073450-BZ), and the study was performed in accordance with regulations. Written informed consent was obtained from all participants before enrollment in the study.

### Blood and Urine samples collection

Venous blood samples were drawn from an antecubital vein and collected in heparinized tubes (Becton Dickinson Company, UK), centrifuged and separated. Plasma was stored in aliquots at −80 °C until analysis. Urine samples were also stored at −80 °C. Urine samples were collected in a sterile container provided to the participants. All samples were stored in multiple aliquots at −80 °C until assayed.

### ROS detection

A X-band EPR instrument (E-scan-Bruker BioSpin, GmbH, MA) was used for determination of ROS. The instrument is designed to function with very low concentrations of paramagnetic species in small (50 μL) samples. For each recruited participant, the ROS production rate was determined at rest by means of a recently implemented EPR method^17^. Determination involved analysing 50 μL plasma samples treated with a CMH (1-hydroxy-3-methoxycarbonyl-2,2,5,5-tetramethylpyrrolidine) probe solution (1:1), in order to transform ROS into more stable radical species that are EPR detectable. 50 μL of the obtained solution was then put in a glass EPR capillary tube (Noxygen Science Transfer & Diagnostics, Germany) that was placed inside the cavity of the E-scan spectrometer for data acquisition (Fig. 3). Acquisition parameters were inclusive of a microwave frequency of 9.652 GHz; modulation frequency of 86 kHz; modulation amplitude of 2.28 G; sweep width of 60 G, microwave power of 21.90 mW, number of scans was 10; and receiver gain was equivalent to 3.17·10^1^. Sample temperature was first stabilised and then kept at 37 °C by the temperature and gas controller Bio III unit, interfaced to the spectrometer. Spectra were recorded and analysed using the Win EPR software (2.11 version) supplied by Bruker. EPR measurements allowed us to obtain a relative quantitative determination of ROS production rate in samples. All data were, in turn, converted into absolute concentration (μmol∙min^−1^) by adopting CP^●^ (3-Carboxy-2,2,5,5-tetramethyl-1-pyrrolidinyloxy) stable radical as an external reference.

### Oxidative damage assessment

Immuno and enzymatic determinations: all samples were assessed by immuno and/or enzymatic methods using a microplate reader spectrophotometer (Infinite M200, Tecan, Austria). All sample determinations were assessed in duplicate and the inter-assay coefficient of variation was within the range indicated by the manufacturer.

#### Antioxidant Capacity (TAC)

Plasma TAC was measured by an enzymatic kit (Cayman Chemical, USA). This assay is based on the ability of antioxidants present in the plasma to inhibit the oxidation of 2,2′-azinobis (3-ethylbenzithiazoline) sulfonic acid (ABTS) to the radical cation ABTS^+^ by a peroxidase. The amount of the produced ABTS^+^ has been assessed by measuring the absorbance signals at 750 nm. The antioxidant concentration is proportional to the suppression of the absorbance signal. TAC was evaluated by a *Trolox* (6-hydroxy-2,5,7,8-tetramethylchroman-2-carboxylic acid) standard curve and was expressed as trolox-equivalent antioxidant capacity concentration (mM).

#### 8-isoprostane (8-isoPGF2α)

8-isoPGF2α concentrations were measured using a commercially available enzyme immunoassay kit (Cayman Chemical, USA). Briefly, 50 μl of urine samples were placed in a 96-well plate that was pre-coated with mouse monoclonal antibody. Thereafter, 50 μl of 8-iso PGF2α-tracer and 8-isoPGF2α-antiserum were added into each well and incubated for 18 h at 4 °C. After washing with buffer, 200 μl of Ellman’s reagent containing the substrate of acetylcholinesterase was added. The plates were read at a wavelength between 405 and 420 nm. The lower detection limit of the assay was 0.8 pg·mL^−1^. The sample 8-isoPGF2α concentrations were determined using 8-isoPGF2α standard curve.

#### Thiobarbituric acid-reactive substances (TBARS)

The measurement of TBARS is a well-established method to detect lipid peroxidation. A TBARS assay kit (Cayman Chemical, USA) was employed, which allows a rapid photometric detection of the thiobarbituric acid malondialdehyde (TBAMDA) adduct at 532 nm. A linear calibration curve was computed from pure MDA-containing reactions.

### ONSD ultrasonography

Ultrasound examinations (MyLabGold30, Esaote, Italy) were performed as previously described^18^ with a linear transducer (3–11 MHz). The papilla and optic nerve were depicted longitudinally in an axial plane with the probe on the temporal part of the upper eyelid. All ultrasound examinations were performed by the same investigator (blinded to medical examinations). The diameter was measured 3 mm behind the papilla by another investigator. Mean ONSD was calculated per eye (3 measurements) and per participant (mean of right and left eyes) (Fig. 3). Interobserver agreement for ONSD measurements previously reported in our group was high (concordance coefficient 0.88; 95% confidence interval 0.84–0.91)^15^.

### Statistical Analysis

ANOVA for repeated measures followed by paired samples *t*-test (with Bonferroni correction) was used for the analysis of changes over time. When variables were not normally distributed, the Friedman and Wilcoxon signed-rank test were used. For 8-isoPGF2α an estimated value of the general population^32^ was used as the baseline value. ROS, TBARS and TAC were correlated with ONSD, SpO_2_ and LLS by means of a model including subject as a random effect, first without time and then adding time as fixed effect. In the correlation analysis of ROS, TBARS and TAC with ONSD and time significance of age, BMI, gender, percentage body fat and smoker (yes/no) was tested. Correlation coefficients were obtained by using the square root of the proportion of variation in the outcome variable that is explained by the covariate/factor^33^; they were assessed for both absolute values (correlation of level) and relative increase from previous time-points (correlation of differences). A logarithmic transformation was used to obtain a normal distribution of absolute values of ROS (lnROS). A multivariate analysis of factors associated with ONSD was performed by means of generalized estimating equations for all measurements up to 24 h: the model including time, AMS and interaction of AMS and HR with time^15^ was taken as a reference and ROS, TBARS, TAC and their interaction with time were added to the model to test their significance. SPSS version 22.0.0.0 statistical software (IBM Corp., Armonk, NY) was used; p < 0.05 (two sided) was considered statistically significant.

## Additional Information

**How to cite this article**: Strapazzon, G. *et al*. Oxidative stress response to acute hypobaric hypoxia and its association with indirect measurement of increased intracranial pressure: a field study. *Sci. Rep.* **6**, 32426; doi: 10.1038/srep32426 (2016).

## Figures and Tables

**Table 1:** ROS, TBARS, TAC, 8-isoPGF2α, clinical parameters and ONSD at baseline and during exposure to 3830 m.

	BL	9 h	24 h	72 h	Repeated measures ANOVA p-value	Paired samples *t*-test p-value after Bonferroni correction					
						9 h *vs.* BL	24 h *vs.* BL	72 h *vs*. BL	24 h *vs.*9 h	72 h *vs.* 9 h	72 h *vs.* 24 h
Participants [males], n	16 [12]	16 [12]	16 [12]	15 [11]							
ROS, (μmol∙min^−1^)*	0.17 (0.02)	0.31 (0.16)	0.41 (0.20)	0.26 (0.06)	<0.001	0.030	0.004	0.018	0.046	1.000	0.017
TBARS, (μM)	7.98 (1.10)	10.16 (1.07)	11.96 (2.25)	9.19 (2.00)	<0.001	0.005	<0.001	0.209	0.107	1.000	0.038
TAC, (mM)	2.02 (0.37)	1.73 (0.31)	1.20 (0.28)	1.49 (0.34)	<0.001	0.018	<0.001	0.005	0.460	0.122	0.149
8-isoPGF2α, (ng∙mg^−1^ creatinine)**	0.40 (0.25)	0.60 (0.19)	0.74 (0.17)	0.68 (0.13)	0.015	0.006	<0.001	<0.001	0.011	1.000	1.000
LLS*	0.00 (0.00)	0.53 (0.74)	1.38 (2.42)	0.40 (0.51)	0.052	0.380	0.203	0.273	1.000	1.000	0.353
SpO_2_*, %	98.63 (1.54)	86.85 (5.51)	87.25 (4.75)	91.53 (2.17)	<0.001	0.009	0.003	0.004	1.000	0.022	0.013
HR, bpm	63.00 (8.44)	84.93 (17.47)	82.63 (10.68)	76.27 (14.73)	<0.001	0.001	<0.001	0.005	1.000	0.022	0.491
ONSD, mm	5.42 (0.29)	6.30 (0.37)	6.33 (0.39)	6.11 (0.31)	<0.001	<0.001	<0.001	<0.001	1.000	0.652	0.038

Data are shown as mean (standard deviation).

8-isoPGF2α, 8-isoprostanes; BL, baseline; bpm, beats per minute; HR, heart rate; LLS, Lake Louise Score; mm, millimeters; ONSD, optic nerve sheath diameter; rpm, rate per minute; ROS, reactive oxygen species; SpO_2_, oxygen saturation; TAC, total antioxidant capacity; TBARS, thiobarbituric acid-reactive substances.

^*^Friedman test instead of repeated measures ANOVA and Wilcoxon signed ranks test instead of paired samples t-test.

^**^Value at BL taken from an average population.

**Table 2:** Estimated correlation coefficients† of ROS, TBARS and TAC with ONSD, SpO_2_ and LLS.

Time included in the model	Covariate/factor	Absolute values						Relative increase from previous time-point					
		ln(ROS)		TBARS		TAC		ROS		TBARS		TAC	
		correlation	p-value	correlation	p-value	correlation	p-value	correlation	p-value	correlation	p-value	correlation	p-value
no	ONSD	0.713	<0.001	0.606	<0.001	0.741	<0.001	0.444	0.065	0.462	0.054	0.382	0.177
yes	time	0.693	<0.001	0.621	0.001	0.488	0.036	0.618	0.035	0.681	0.013	0.521	0.205
	ONSD	0.101	0.559	0.075	0.662	0.193	0.281	0.235	0.381	0.106	0.695	0.133	0.680
no	SpO_2_	0.671	<0.001	0.556	<0.001	0.630	<0.001	0.457	0.057	0.406	0.095	0.327	0.254
yes	time	0.756	<0.001	0.676	<0.001	0.647	0.001	0.580	0.057	0.708	0.008	0.540	0.179
	SpO_2_	0.295	0.081	0.203	0.235	0.132	0.465	0.018	0.946	0.149	0.581	0.023	0.942
no	LLS#	0.430	0.010	0.332	0.052	0.286	0.113	0.103	0.686	0.183	0.468	0.044	0.882
yes	time	0.823	<0.001	0.728	<0.001	0.800	<0.001	0.687	0.011	0.748	0.003	0.609	0.098
	LLS#	0.107	0.560	0.031	0.868	0.229	0.231	0.080	0.768	0.003	0.990	0.096	0.767

LLS, Lake Louise Score; ONSD, optic nerve sheath diameter; ROS, reactive oxygen species; SpO_2_, oxygen saturation; TAC, total antioxidant capacity; TBARS, thiobarbituric acid-reactive substances.

^†^Correlation coefficients are given as sqrt (sum of squares) formula from ANOVA with subject as random effect, ONSD, SpO_2_ or LLS as covariate and respective parameter as dependent variable.

^#^In groups: 0, ≥1.

**Table 3:** Regression analysis (generalized estimating equations) of factors associated with ONSD.

Effect	p-value (effect)	Parameter	B	95% CI	p-value (parameter)
Intercept	<0.001	Intercept	8.389	7.530, 9.248	<0.001
Time	0.003	Time = baseline	−2.112	−3.355, −0.869	0.001
		Time = 9 h	−1.237	−1.967, −0.506	0.001
		Time = 24 h	0.000		
AMS	<0.001	AMS = no	−0.697	−0.897, −0.497	<0.001
Time*AMS	a				
Time*HR	0.003	Time = baseline*HR (bpm)	−0.004	−0.017, 0.010	0.569
		Time = 9 h*HR (bpm)	−0.003	−0.006, 0.001	0.135
		Time = 24 h*HR (bpm)	−0.017	−0.029, −0.006	0.002
ROS	a				
Time*ROS	a				
TBARS	a				
Time*TBARS	a				
TAC	a				
Time*TAC	a				

AMS, acute mountain sickness (Lake Louise score ≥3 with headache); B, estimated coefficient; CI, confidence interval; HR, heart rate; ROS, reactive oxygen species; TAC, total antioxidant capacity; TBARS, thiobarbituric acid-reactive substances.

^a^Not significant (parameter eliminated during the stepwise procedure).
